# 5-(4-Chloro­phen­oxy)-1-methyl-3-tri­fluoro­methyl-1*H*-pyrazole-4-carbaldehyde *O*-[(2-chloro­pyridin-5-yl)meth­yl]oxime

**DOI:** 10.1107/S1600536811046459

**Published:** 2011-11-09

**Authors:** Hong Dai, Peng-Fei Zhu, Yu-Jun Zhu, Jian-Xin Fang, Yu-Jun Shi

**Affiliations:** a College of Chemistry and Chemical Engineering, Nantong University, Nantong 226019, People’s Republic of China; b State Key Laboratory and Institute of Elemento-Organic Chemistry, Nankai University, Tianjin 300071, People’s Republic of China

## Abstract

In the title mol­ecule, C_18_H_13_Cl_2_F_3_N_4_O_2_, the intra­molecular distance between the centroids of the benzene and pyridine rings is 3.953 (3) Å, and the trifluoro­methyl group is rotationally disordered over two orientations in a 0.678 (19):0.322 (19) ratio. The crystal packing exhibits weak inter­molecular C—H⋯F inter­actions.

## Related literature

For the crystal structure of a related pyrazole oxime studied recently by our group, see: Dai *et al.* (2011 ▶).
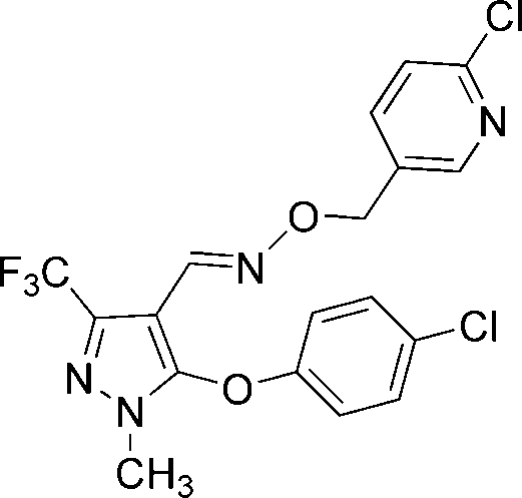


## Experimental

### 

#### Crystal data

C_18_H_13_Cl_2_F_3_N_4_O_2_
*M* *_r_* = 445.22
Monoclinic, 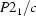

*a* = 12.269 (3) Å
*b* = 10.443 (2) Å
*c* = 15.702 (3) Å
β = 108.93 (3)°
*V* = 1902.9 (7) Å^3^
*Z* = 4
Mo *K*α radiation
μ = 0.39 mm^−1^
*T* = 113 K
0.14 × 0.10 × 0.08 mm

#### Data collection

Rigaku Saturn diffractometer
Absorption correction: multi-scan (*CrystalClear*; Rigaku, 2008 ▶) *T*_min_ = 0.947, *T*_max_ = 0.969
10759 measured reflections
3356 independent reflections
2845 reflections with *I* > 2σ(*I*)
*R*_int_ = 0.041

#### Refinement

*R*[*F* ^2^ > 2σ(*F* ^2^)] = 0.035
*wR*(*F* ^2^) = 0.090
*S* = 1.07
3356 reflections
291 parameters
66 restraints
H-atom parameters constrained
Δρ_max_ = 0.29 e Å^−3^
Δρ_min_ = −0.26 e Å^−3^

### 

Data collection: *CrystalClear* (Rigaku, 2008 ▶); cell refinement: *CrystalClear* (Rigaku, 2008 ▶); data reduction: *CrystalClear*; program(s) used to solve structure: *SHELXS97* (Sheldrick, 2008 ▶); program(s) used to refine structure: *SHELXL97* (Sheldrick, 2008 ▶); molecular graphics: *SHELXTL* (Sheldrick, 2008 ▶); software used to prepare material for publication: *SHELXTL*.

## Supplementary Material

Crystal structure: contains datablock(s) global, I. DOI: 10.1107/S1600536811046459/cv5190sup1.cif

Structure factors: contains datablock(s) I. DOI: 10.1107/S1600536811046459/cv5190Isup2.hkl

Supplementary material file. DOI: 10.1107/S1600536811046459/cv5190Isup3.cml

Additional supplementary materials:  crystallographic information; 3D view; checkCIF report

## Figures and Tables

**Table 1:** Hydrogen-bond geometry (Å, °)

*D*—H⋯*A*	*D*—H	H⋯*A*	*D*⋯*A*	*D*—H⋯*A*
C5—H5*C*⋯F3^i^	0.96	2.55	3.488 (7)	165
C11—H11⋯F3′^ii^	0.93	2.56	3.358 (14)	144

Symmetry codes: (i) 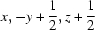; (ii) 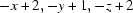.

**Fig. 1.**
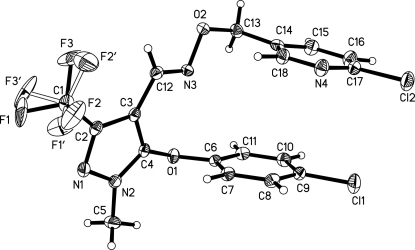
The molecular structure of (I) showing the atomic numbering and 50% probabilty displacement ellipsoids.

## supplementary crystallographic information

### Comment

As a continuation of our structural study of pyrazole oximes (Dai *et al.*, 2011), we report here the crystal structure of the title compound (I).

In (I) (Fig. 1), all bonds lengths and angles are similar to those observed in the related compound (Dai *et al.*, 2011). The dihedral angles between the planes of the pyridyl and pyrazole rings, and between the benzene and the pyrazole rings are 91.0 (3)° and 95.8 (3)°, respectively. The crystal packing displays weak intermolecular C—H···F interactions (Table 1).

### Experimental

To a well stirred solution of 1-methyl-3-trifluoromethyl-5-(4-chlorophenoxy)- 1*H*-pyrazole-4-carbaldehyde oxime (3 mmol) and 2-chloro-5-chloromethylpyridine (3.6 mmol) in 40 ml of anhydrous DMF, was added powdered potassium carbonate (7.5 mmol). The resulting solution was heated to 363 K for 10 h and cooled to room temperature. The mixture was poured into water (180 ml) and extracted with dichloromethane (4 * 50 ml). The organic layer was washed with saturated brine (3 * 30 ml) and dried over anhydrous sodium sulfate. The solvent was evaporated under reduced pressure, the residue was separated by column chromatography on silica gel with petroleum ether/ethyl acetate (10:1 *v*/*v*) as eluent, and then recrystallized from ethyl acetate to give a colourless crystal.

### Refinement

H atoms were placed in calculated positions, with C–H = 0.93 - 0.97 ° A, and refined as riding, with *U*_iso_(H) = 1.2-1.5 *U*_eq_(C). The trifluoromethyl was treated as disordered over two orientations. The displacement parameters of atoms F1, F2, F3, F1', F2' and F3' were restrained to behave approximately isotropic.

### Crystal data

**Table:**

C_18_H_13_Cl_2_F_3_N_4_O_2_	*F*(000) = 904
*M**_r_* = 445.22	*D*_x_ = 1.554 Mg m^−^^3^
Monoclinic, *P*2_1_/*c*	Mo *K*α radiation, λ = 0.71073 Å
Hall symbol: -P 2ybc	Cell parameters from 4362 reflections
*a* = 12.269 (3) Å	θ = 1.8–27.9°
*b* = 10.443 (2) Å	µ = 0.39 mm^−^^1^
*c* = 15.702 (3) Å	*T* = 113 K
β = 108.93 (3)°	Monoclinic, colourless
*V* = 1902.9 (7) Å^3^	0.14 × 0.10 × 0.08 mm
*Z* = 4	

### Data collection

**Table:**

Rigaku Saturn diffractometer	3356 independent reflections
Radiation source: rotating anode	2845 reflections with *I* > 2σ(*I*)
confocal	*R*_int_ = 0.041
ω scans	θ_max_ = 25.0°, θ_min_ = 2.4°
Absorption correction: multi-scan (*CrystalClear*; Rigaku, 2008)	*h* = −14→14
*T*_min_ = 0.947, *T*_max_ = 0.969	*k* = −12→12
10759 measured reflections	*l* = −14→18

### Refinement

**Table:**

Refinement on *F*^2^	Primary atom site location: structure-invariant direct methods
Least-squares matrix: full	Secondary atom site location: difference Fourier map
*R*[*F*^2^ > 2σ(*F*^2^)] = 0.035	Hydrogen site location: inferred from neighbouring sites
*wR*(*F*^2^) = 0.090	H-atom parameters constrained
*S* = 1.07	*w* = 1/[σ^2^(*F*_o_^2^) + (0.0531*P*)^2^] where *P* = (*F*_o_^2^ + 2*F*_c_^2^)/3
3356 reflections	(Δ/σ)_max_ = 0.003
291 parameters	Δρ_max_ = 0.29 e Å^−^^3^
66 restraints	Δρ_min_ = −0.26 e Å^−^^3^

### Special details

**Table:**

Geometry. All e.s.d.'s (except the e.s.d. in the dihedral angle between two l.s. planes) are estimated using the full covariance matrix. The cell e.s.d.'s are taken into account individually in the estimation of e.s.d.'s in distances, angles and torsion angles; correlations between e.s.d.'s in cell parameters are only used when they are defined by crystal symmetry. An approximate (isotropic) treatment of cell e.s.d.'s is used for estimating e.s.d.'s involving l.s. planes.
Refinement. Refinement of *F*^2^ against ALL reflections. The weighted *R*-factor *wR* and goodness of fit *S* are based on *F*^2^, conventional *R*-factors *R* are based on *F*, with *F* set to zero for negative *F*^2^. The threshold expression of *F*^2^ > σ(*F*^2^) is used only for calculating *R*-factors(gt) *etc*. and is not relevant to the choice of reflections for refinement. *R*-factors based on *F*^2^ are statistically about twice as large as those based on *F*, and *R*- factors based on ALL data will be even larger.

### Fractional atomic coordinates and isotropic or equivalent isotropic displacement parameters (Å^2^)

**Table:**

	*x*	*y*	*z*	*U*_iso_*/*U*_eq_	Occ. (<1)
Cl1	0.42312 (4)	0.81673 (5)	1.05301 (4)	0.03177 (16)	
Cl2	0.24719 (4)	0.78497 (5)	0.81487 (3)	0.02958 (16)	
F1	1.0291 (4)	0.1147 (5)	1.0472 (5)	0.0485 (14)	0.678 (19)
F2	0.8486 (6)	0.0969 (7)	1.0153 (6)	0.0436 (15)	0.678 (19)
F3	0.9173 (8)	0.1880 (6)	0.9233 (3)	0.0509 (13)	0.678 (19)
F1'	0.8773 (14)	0.0850 (14)	1.0406 (10)	0.040 (3)	0.322 (19)
F2'	0.8654 (15)	0.1804 (12)	0.9205 (6)	0.052 (3)	0.322 (19)
F3'	1.0278 (9)	0.1329 (11)	1.0091 (13)	0.054 (3)	0.322 (19)
O1	0.89000 (10)	0.62356 (11)	1.10140 (8)	0.0181 (3)	
O2	0.76286 (11)	0.60323 (12)	0.81549 (8)	0.0197 (3)	
N1	0.97610 (13)	0.30373 (14)	1.14348 (10)	0.0184 (3)	
N2	0.96106 (13)	0.42594 (15)	1.16558 (10)	0.0177 (3)	
N3	0.80499 (12)	0.57770 (15)	0.90895 (9)	0.0180 (3)	
N4	0.43641 (14)	0.66049 (15)	0.83015 (11)	0.0229 (4)	
C1	0.92933 (16)	0.17597 (18)	1.00931 (12)	0.0217 (4)	
C2	0.92895 (15)	0.30085 (17)	1.05422 (12)	0.0173 (4)	
C3	0.88375 (15)	0.41957 (17)	1.01704 (12)	0.0163 (4)	
C4	0.90697 (14)	0.49692 (17)	1.09258 (11)	0.0159 (4)	
C5	1.00381 (17)	0.4675 (2)	1.25933 (11)	0.0254 (5)	
H5A	0.9811	0.5546	1.2635	0.038*	
H5B	1.0863	0.4615	1.2812	0.038*	
H5C	0.9721	0.4137	1.2950	0.038*	
C6	0.77559 (15)	0.66348 (17)	1.08527 (11)	0.0171 (4)	
C7	0.69070 (16)	0.58030 (18)	1.09125 (11)	0.0189 (4)	
H7	0.7065	0.4937	1.1029	0.023*	
C8	0.58119 (17)	0.62885 (18)	1.07952 (12)	0.0206 (4)	
H8	0.5226	0.5744	1.0827	0.025*	
C9	0.55957 (17)	0.75740 (18)	1.06322 (12)	0.0204 (4)	
C10	0.64499 (16)	0.84005 (18)	1.05618 (12)	0.0220 (4)	
H10	0.6293	0.9266	1.0444	0.026*	
C11	0.75420 (17)	0.79174 (17)	1.06702 (12)	0.0193 (4)	
H11	0.8123	0.8455	1.0620	0.023*	
C12	0.83287 (15)	0.46034 (18)	0.92371 (12)	0.0177 (4)	
H12	0.8212	0.4026	0.8765	0.021*	
C13	0.72665 (16)	0.73399 (18)	0.80495 (12)	0.0203 (4)	
H13A	0.7263	0.7643	0.7465	0.024*	
H13B	0.7817	0.7853	0.8505	0.024*	
C14	0.60834 (16)	0.75256 (17)	0.81277 (11)	0.0173 (4)	
C15	0.55696 (17)	0.87257 (19)	0.80193 (13)	0.0232 (4)	
H15	0.5971	0.9441	0.7929	0.028*	
C16	0.44578 (17)	0.88534 (19)	0.80457 (13)	0.0250 (5)	
H16	0.4099	0.9649	0.7984	0.030*	
C17	0.39031 (16)	0.77536 (19)	0.81674 (12)	0.0211 (4)	
C18	0.54451 (16)	0.65150 (18)	0.82871 (12)	0.0211 (4)	
H18	0.5794	0.5714	0.8392	0.025*	

### Atomic displacement parameters (Å^2^)

**Table:**

	*U*^11^	*U*^22^	*U*^33^	*U*^12^	*U*^13^	*U*^23^
Cl1	0.0236 (3)	0.0318 (3)	0.0403 (3)	0.0106 (2)	0.0108 (2)	0.0023 (2)
Cl2	0.0196 (3)	0.0357 (3)	0.0373 (3)	0.0061 (2)	0.0147 (2)	0.0090 (2)
F1	0.0318 (16)	0.0334 (17)	0.065 (3)	0.0189 (13)	−0.0060 (15)	−0.0266 (18)
F2	0.038 (2)	0.030 (2)	0.076 (4)	−0.0155 (17)	0.036 (2)	−0.021 (2)
F3	0.108 (4)	0.0256 (13)	0.0330 (16)	0.004 (3)	0.042 (2)	−0.0057 (12)
F1'	0.076 (7)	0.014 (3)	0.043 (5)	−0.016 (4)	0.037 (5)	−0.007 (3)
F2'	0.092 (7)	0.026 (3)	0.030 (3)	0.013 (5)	0.005 (4)	−0.008 (2)
F3'	0.029 (3)	0.047 (4)	0.103 (7)	−0.010 (3)	0.044 (4)	−0.041 (5)
O1	0.0169 (7)	0.0131 (6)	0.0238 (7)	−0.0001 (5)	0.0061 (5)	−0.0023 (5)
O2	0.0201 (7)	0.0238 (7)	0.0148 (6)	0.0045 (6)	0.0051 (5)	0.0026 (5)
N1	0.0178 (8)	0.0160 (8)	0.0231 (8)	0.0020 (7)	0.0087 (7)	0.0020 (6)
N2	0.0188 (8)	0.0169 (8)	0.0191 (8)	0.0020 (7)	0.0087 (7)	0.0009 (6)
N3	0.0167 (8)	0.0237 (9)	0.0135 (7)	0.0028 (7)	0.0049 (6)	0.0032 (7)
N4	0.0208 (9)	0.0206 (9)	0.0295 (9)	0.0007 (7)	0.0115 (7)	0.0026 (7)
C1	0.0201 (11)	0.0190 (10)	0.0277 (10)	−0.0003 (9)	0.0100 (9)	0.0008 (8)
C2	0.0151 (9)	0.0172 (10)	0.0229 (9)	−0.0008 (8)	0.0107 (8)	−0.0005 (8)
C3	0.0144 (9)	0.0161 (9)	0.0204 (9)	0.0002 (8)	0.0083 (8)	0.0001 (7)
C4	0.0132 (9)	0.0149 (9)	0.0210 (9)	0.0006 (7)	0.0074 (8)	0.0004 (7)
C5	0.0309 (12)	0.0278 (11)	0.0167 (9)	−0.0003 (9)	0.0065 (9)	0.0000 (8)
C6	0.0175 (10)	0.0197 (10)	0.0144 (9)	0.0025 (8)	0.0055 (8)	−0.0018 (7)
C7	0.0238 (10)	0.0138 (9)	0.0204 (9)	0.0007 (8)	0.0090 (8)	−0.0010 (8)
C8	0.0212 (10)	0.0208 (10)	0.0216 (9)	−0.0034 (8)	0.0094 (8)	−0.0014 (8)
C9	0.0198 (11)	0.0232 (10)	0.0179 (9)	0.0048 (8)	0.0058 (8)	−0.0020 (8)
C10	0.0288 (11)	0.0149 (9)	0.0221 (9)	0.0026 (8)	0.0079 (9)	0.0014 (8)
C11	0.0239 (10)	0.0151 (9)	0.0200 (9)	−0.0030 (8)	0.0086 (8)	−0.0012 (7)
C12	0.0155 (9)	0.0202 (10)	0.0178 (9)	−0.0014 (8)	0.0057 (8)	−0.0029 (8)
C13	0.0200 (11)	0.0200 (10)	0.0211 (9)	0.0015 (8)	0.0069 (8)	0.0051 (8)
C14	0.0189 (10)	0.0193 (10)	0.0135 (8)	−0.0018 (8)	0.0048 (8)	0.0012 (7)
C15	0.0242 (11)	0.0198 (10)	0.0277 (10)	−0.0022 (9)	0.0114 (9)	0.0028 (8)
C16	0.0280 (11)	0.0188 (10)	0.0311 (11)	0.0058 (9)	0.0136 (9)	0.0060 (8)
C17	0.0169 (10)	0.0275 (11)	0.0199 (9)	0.0022 (9)	0.0075 (8)	0.0023 (8)
C18	0.0208 (10)	0.0187 (10)	0.0248 (10)	0.0022 (8)	0.0090 (8)	0.0016 (8)

### Geometric parameters (Å, °)

**Table:**

Cl1—C9	1.743 (2)	C5—H5B	0.9600
Cl2—C17	1.7496 (19)	C5—H5C	0.9600
F1—C1	1.338 (4)	C6—C11	1.377 (3)
F2—C1	1.316 (5)	C6—C7	1.383 (3)
F3—C1	1.317 (4)	C7—C8	1.391 (3)
F1'—C1	1.324 (9)	C7—H7	0.9300
F2'—C1	1.362 (8)	C8—C9	1.376 (3)
F3'—C1	1.291 (7)	C8—H8	0.9300
O1—C4	1.353 (2)	C9—C10	1.389 (3)
O1—C6	1.406 (2)	C10—C11	1.390 (3)
O2—N3	1.4141 (18)	C10—H10	0.9300
O2—C13	1.429 (2)	C11—H11	0.9300
N1—C2	1.332 (2)	C12—H12	0.9300
N1—N2	1.351 (2)	C13—C14	1.509 (2)
N2—C4	1.345 (2)	C13—H13A	0.9700
N2—C5	1.459 (2)	C13—H13B	0.9700
N3—C12	1.273 (2)	C14—C18	1.384 (3)
N4—C17	1.314 (2)	C14—C15	1.388 (3)
N4—C18	1.337 (2)	C15—C16	1.384 (3)
C1—C2	1.483 (3)	C15—H15	0.9300
C2—C3	1.405 (3)	C16—C17	1.380 (3)
C3—C4	1.386 (2)	C16—H16	0.9300
C3—C12	1.458 (2)	C18—H18	0.9300
C5—H5A	0.9600		
			
C4—O1—C6	116.69 (14)	H5B—C5—H5C	109.5
N3—O2—C13	107.19 (13)	C11—C6—C7	121.89 (17)
C2—N1—N2	104.05 (14)	C11—C6—O1	115.95 (16)
C4—N2—N1	111.86 (14)	C7—C6—O1	122.10 (16)
C4—N2—C5	127.74 (16)	C6—C7—C8	118.58 (17)
N1—N2—C5	120.38 (15)	C6—C7—H7	120.7
C12—N3—O2	110.88 (14)	C8—C7—H7	120.7
C17—N4—C18	116.05 (16)	C9—C8—C7	119.98 (18)
F3'—C1—F2	120.6 (6)	C9—C8—H8	120.0
F3'—C1—F3	79.8 (6)	C7—C8—H8	120.0
F2—C1—F3	107.2 (4)	C8—C9—C10	121.05 (18)
F3'—C1—F1'	108.4 (9)	C8—C9—Cl1	119.03 (15)
F2—C1—F1'	19.8 (7)	C10—C9—Cl1	119.91 (15)
F3—C1—F1'	122.8 (7)	C9—C10—C11	119.14 (18)
F3'—C1—F1	27.4 (6)	C9—C10—H10	120.4
F2—C1—F1	105.6 (4)	C11—C10—H10	120.4
F3—C1—F1	106.5 (3)	C6—C11—C10	119.32 (17)
F1'—C1—F1	88.5 (7)	C6—C11—H11	120.3
F3'—C1—F2'	103.7 (5)	C10—C11—H11	120.3
F2—C1—F2'	84.1 (6)	N3—C12—C3	117.79 (16)
F3—C1—F2'	27.1 (6)	N3—C12—H12	121.1
F1'—C1—F2'	102.5 (8)	C3—C12—H12	121.1
F1—C1—F2'	127.7 (6)	O2—C13—C14	112.62 (15)
F3'—C1—C2	117.1 (5)	O2—C13—H13A	109.1
F2—C1—C2	113.5 (4)	C14—C13—H13A	109.1
F3—C1—C2	112.8 (3)	O2—C13—H13B	109.1
F1'—C1—C2	112.2 (8)	C14—C13—H13B	109.1
F1—C1—C2	110.6 (2)	H13A—C13—H13B	107.8
F2'—C1—C2	111.6 (5)	C18—C14—C15	116.70 (17)
N1—C2—C3	113.29 (16)	C18—C14—C13	122.17 (17)
N1—C2—C1	116.93 (16)	C15—C14—C13	121.11 (17)
C3—C2—C1	129.77 (17)	C16—C15—C14	119.73 (18)
C4—C3—C2	102.40 (15)	C16—C15—H15	120.1
C4—C3—C12	126.31 (17)	C14—C15—H15	120.1
C2—C3—C12	131.13 (16)	C17—C16—C15	117.33 (18)
N2—C4—O1	120.07 (15)	C17—C16—H16	121.3
N2—C4—C3	108.40 (15)	C15—C16—H16	121.3
O1—C4—C3	131.42 (16)	N4—C17—C16	125.19 (17)
N2—C5—H5A	109.5	N4—C17—Cl2	115.64 (14)
N2—C5—H5B	109.5	C16—C17—Cl2	119.18 (15)
H5A—C5—H5B	109.5	N4—C18—C14	124.88 (18)
N2—C5—H5C	109.5	N4—C18—H18	117.6
H5A—C5—H5C	109.5	C14—C18—H18	117.6
			
C2—N1—N2—C4	−0.33 (19)	C12—C3—C4—O1	0.4 (3)
C2—N1—N2—C5	−179.09 (15)	C4—O1—C6—C11	−160.71 (15)
C13—O2—N3—C12	176.22 (14)	C4—O1—C6—C7	22.0 (2)
N2—N1—C2—C3	0.4 (2)	C11—C6—C7—C8	−0.8 (3)
N2—N1—C2—C1	−178.44 (14)	O1—C6—C7—C8	176.26 (15)
F3'—C1—C2—N1	−69.1 (10)	C6—C7—C8—C9	−0.6 (3)
F2—C1—C2—N1	78.6 (5)	C7—C8—C9—C10	1.5 (3)
F3—C1—C2—N1	−159.1 (5)	C7—C8—C9—Cl1	−177.60 (14)
F1'—C1—C2—N1	57.1 (8)	C8—C9—C10—C11	−0.9 (3)
F1—C1—C2—N1	−40.0 (5)	Cl1—C9—C10—C11	178.20 (13)
F2'—C1—C2—N1	171.6 (10)	C7—C6—C11—C10	1.4 (3)
F3'—C1—C2—C3	112.2 (10)	O1—C6—C11—C10	−175.83 (15)
F2—C1—C2—C3	−100.0 (5)	C9—C10—C11—C6	−0.6 (3)
F3—C1—C2—C3	22.2 (5)	O2—N3—C12—C3	176.84 (14)
F1'—C1—C2—C3	−121.5 (8)	C4—C3—C12—N3	−1.1 (3)
F1—C1—C2—C3	141.4 (5)	C2—C3—C12—N3	−175.70 (18)
F2'—C1—C2—C3	−7.1 (10)	N3—O2—C13—C14	−81.09 (17)
N1—C2—C3—C4	−0.4 (2)	O2—C13—C14—C18	0.2 (2)
C1—C2—C3—C4	178.33 (17)	O2—C13—C14—C15	−178.18 (16)
N1—C2—C3—C12	175.19 (17)	C18—C14—C15—C16	−2.0 (3)
C1—C2—C3—C12	−6.1 (3)	C13—C14—C15—C16	176.44 (17)
N1—N2—C4—O1	−176.49 (14)	C14—C15—C16—C17	−1.0 (3)
C5—N2—C4—O1	2.2 (3)	C18—N4—C17—C16	−2.2 (3)
N1—N2—C4—C3	0.12 (19)	C18—N4—C17—Cl2	177.86 (13)
C5—N2—C4—C3	178.76 (16)	C15—C16—C17—N4	3.3 (3)
C6—O1—C4—N2	−111.17 (17)	C15—C16—C17—Cl2	−176.79 (14)
C6—O1—C4—C3	73.1 (2)	C17—N4—C18—C14	−1.2 (3)
C2—C3—C4—N2	0.13 (18)	C15—C14—C18—N4	3.3 (3)
C12—C3—C4—N2	−175.71 (16)	C13—C14—C18—N4	−175.16 (17)
C2—C3—C4—O1	176.22 (17)		

### Hydrogen-bond geometry (Å, °)

**Table:**

*D*—H···*A*	*D*—H	H···*A*	*D*···*A*	*D*—H···*A*
C5—H5C···F3^i^	0.96	2.55	3.488 (7)	165
C11—H11···F3'^ii^	0.93	2.56	3.358 (14)	144

Symmetry codes: (i) *x*, −*y*+1/2, *z*+1/2; (ii) −*x*+2, −*y*+1, −*z*+2.
